# *Pseudomonas aeruginosa* in the ICU: prevalence, resistance profile, and antimicrobial consumption

**DOI:** 10.1590/0037-8682-0498-2018

**Published:** 2019-12-20

**Authors:** Ághata Cardoso da Silva Ribeiro, Márcia Terezinha Lonardoni Crozatti, Adilson Aderito da Silva, Rodrigo Spineli Macedo, Antonia Maria de Oliveira Machado, Antonio Távora de Albuquerque Silva

**Affiliations:** 1 Universidade Federal de São Paulo, Instituto de Ciências Ambientais, Químicas e Farmacêuticas, Diadema, SP, Brasil.; 2 Universidade Presbiteriana Mackenzie, Centro de Ciências Sociais e Aplicadas, São Paulo, SP, Brasil.; 3 Universidade Federal de São Paulo, Hospital Universitário da UNIFESP/Hospital São Paulo, São Paulo, SP, Brasil.; 4 Universidade Federal de São Paulo. Departamento de Medicina. Laboratório Central do Hospital São Paulo, São Paulo, SP, Brasil.

**Keywords:** Pseudomonas aeruginosa, Antimicrobial agents, Drug resistance

## Abstract

**INTRODUCTION:**

*Pseudomonas aeruginosa* is one of the main pathogens causing infection in intensive care units (ICUs) and usually presents antimicrobial resistance.

**METHODS:**

Data were obtained from ICUs between 2010 and 2013.

**RESULTS:**

*P. aeruginosa* had a prevalence of 14.5% of which 48.7% were multidrug resistant. We observed increasing resistance to carbapenems and polymyxin B and growing consumption of aminoglycosides, meropenem, ceftazidime, and polymyxin B. The regression impact between resistance and consumption was significant with respect to amikacin, imipenem, meropenem, and polymyxin B.

**CONCLUSIONS:**

Monitoring antimicrobial consumption and resistant microorganisms should be reinforced to combat antimicrobial- and multi-drug resistance.

Antimicrobial resistance is a public health concern^1^. Among resistant pathogens, the bacterium *Pseudomonas aeruginosa* is of note because it is often associated with high rates of morbidity and mortality in patients in intensive care units (ICUs)^2^. *P. aeruginosa* is resistant to several antibiotics and has the ability to rapidly develop resistance to new antimicrobials. Moreover, *P. aeruginosa* was the first bacterium to present multidrug-resistant (MDR) phenotypes^3^.

Therefore, it is important to monitor the consumption of antibiotics, especially in a hospital environment, to avoid the development of resistance and improve the therapeutic efficacy of these drugs against *P. aeruginosa*.

To address the above issues, this study aimed to describe the prevalence of resistant and MDR isolates of *P. aeruginosa* in nine ICUs of a university hospital, as well as to describe the consumption of antimicrobial agents using a defined daily dose (DDD) and investigate the relationship between the consumption of and resistance to antibiotics in the nine ICUs.

This was a retrospective descriptive study, conducted from January 2010 to December 2013 in ICUs of the Hospital São Paulo - university hospital (HSP-HU/Unifesp) in the city of São Paulo (Brazil).

For resistance and susceptibility analysis, data from the Clinical Laboratory of the hospital were used. Samples were obtained from patients hospitalized in at least one of the ICUs selected during the study period.

*P. aeruginosa* isolates were considered to be MDR if they showed resistance to three or more classes of antimicrobial agents, with resistance to at least one antibiotic in each class^4^.

Data on the consumption of antimicrobials (in grams) were provided by the Supplies System of the hospital, and the hospitalization rate in the study period was provided by the Hospital Statistics Sector. Antimicrobial agents were classified according to the Anatomical Therapeutic Chemical system, and consumption was measured in DDD per 100 bed-days^5^.

Susceptibility tests performed on samples from anal and rectal swabs for *Klebsiella pneumoniae* carbapenemase surveillance and vancomycin-resistant enterococci surveillance were excluded from the study.

For the organization and analysis of the data, Microsoft® Office Excel for Mac, version 16.27 (Microsoft Corporation) and the Statistical Package for Social Sciences version 20 (IBM Corp, Chicago, IL, USA) were used.

The resistance trend of *P. aeruginosa*, monthly consumption of antimicrobial agents, and the association between antimicrobial agent consumption and resistance of *P. aeruginosa* were analyzed using a linear regression model. The β coefficient indicates the association between a dependent variable and independent variables analyzed by the linear regression model. The significance level was set at 0.05.

The remaining analyses were for normality, linearity, and homoscedasticity. For normality analyses, the Kolmogorov-Smirnov (KS) test (significance>0.05) was performed and all the variables obtained were considered normal, except for the data on consumption of ceftazidime and polymyxin resistance. The homoscedasticity and linearity analyses were conducted by graphical analyses. Homoscedasticity was analyzed using the standardized variables against the standardized predicted values.

This study is part of the project “Prevalent microorganisms in ICU: antimicrobial agents consumption and resistance profiles,” approved by the Research Ethics Committee (opinion 921.500), in accordance with Resolution No. 466 of December 12, 2012, of the National Health Council.

Of 6,473 microorganisms isolated, 939 were identified as *P. aeruginosa* (14.5%). *P. aeruginosa* was the second most prevalent bacterium during the study period, after *Acinetobacter baumannii* (17.3%). The prevalence of *P. aeruginosa* isolates resistant to antimicrobial agents by year is presented in Table 1.

**TABLE 1:** Prevalence of *P. aeruginosa* isolates resistant to the tested antibiotics, based on laboratory findings of patients in nine ICUs from January 2010 to December 2013.

Class / Antimicrobial agent	Total isolates	Total resistant		Resistant isolates							
	(S+I+R)	isolates									
				2010		2011		2012		2013	
	n	N	%	n	%	N	%	n	%	n	%
Aminoglycosides											
Amikacin	925	263	28.4	98	37.3	67	25.5	63	24.0	35	13.3
Streptomycin	11	6	54.6	1	16.7	0	0.0	4	66.7	1	16.7
Gentamicin	938	434	46.3	146	33.6	92	21.2	87	20.0	109	25.1
Tobramycin	53	34	64.2	0	0.0	4	11.8	25	73.5	5	14.7
Carbapenems											
Ertapenem	23	10	43.5	0	0.0	0	0.0	1	10.0	9	90.0
Imipenem	931	477	51.2	127	26.6	116	24.3	108	22.6	126	26.4
Meropenem	930	473	50.9	129	27.3	112	23.7	103	21.8	129	27.3
Cephalosporins											
Cefepime	928	410	44.2	132	32.2	92	22.4	81	19.8	105	25.6
Cefotaxime	26	26	100.0	8	30.8	6	23.1	4	15.4	8	30.8
Cefoxitin	1	1	100.0	0	0.0	0	0.0	0	0.0	1	100.0
Ceftazidime	920	366	39.8	124	33.9	73	19.9	67	18.3	102	27.9
Ceftriaxone	35	32	91.4	2	6.3	1	3.1	5	15.6	24	75.0
Fluoroquinolones											
Ciprofloxacin	928	459	49.5	155	33.8	103	22.4	85	18.5	116	25.3
Levofloxacin	3	3	100.0	2	66.7	0	0.0	0	0.0	1	33.3
Moxifloxacin	2	0	0.0	0	0.0	0	0.0	0	0.0	0	0.0
Norfloxacin	90	49	54.4	27	55.1	12	24.5	7	14.3	3	6.1
Monobactams											
Aztreonam	924	377	40.8	101	26.8	87	23.1	83	22.0	106	28.1
Polymyxins											
Polymyxin B	766	7	0.9	0	0.0	1	14.3	0	0.0	6	85.7
Penicillins											
Amoxicillin	31	28	90.3	1	3.6	1	3.6	3	10.7	23	82.1
Ampicillin	15	12	80.0	2	16.7	1	8.3	6	50.0	3	25.0
Oxacillin	2	2	100.0	2	100.0	0	0.0	0	0.0	0	0.0
Penicillin	9	7	77.8	0	0.0	1	14.3	4	57.1	2	28.6
Piperacillin/Tazobactam	929	334	36.0	130	38.9	83	24.9	58	17.4	63	18.9
Macrolides											
Erythromycin	2	2	100.0	2	100.0	0	0.0	0	0.0	0	0.0
Lincosamides											
Clindamycin	2	2	100.0	2	100.0	0	0.0	0	0.0	0	0.0
Oxazolidinones											
Linezolid	11	0	0.0	0	0.0	0	0.0	0	0.0	0	0.0
Sulfonamides											
Sulfamethoxazole/ Trimethoprim	7	6	85.7	2	33.3	0	0.0	2	33.3	2	33.3
Glycopeptides											
Teicoplanin	10	5	50.0	0	0.0	0	0.0	2	40.0	3	60.0
Vancomycin	11	8	72.7	0	0.0	1	12.5	3	37.5	4	50.0
Tetracyclines											
Tigecycline	17	2	11.8	1	50.0	0	0.0	0	0.0	1	50.0
Others											
Ethambutol	2	0	0.0	0	0.0	0	0.0	0	0.0	0	0.0
Isoniazid	2	0	0.0	2	0.0	0	0.0	0	0.0	0	0.0
Nitrofurantoin	2	1	50.0	0	0.0	0	0.0	0	0.0	1	100.0
Pyrazinamide	2	0	0.0	0	0.0	0	0.0	0	0.0	0	0.0
Rifampicin	4	1	25.0	1	100.0	0	0.0	0	0.0	0	0.0

**I**: intermediate; **R**: resistant; **S**: susceptible.

Increasing resistance of *P. aeruginosa* to imipenem (β=0.419; p=0.003), meropenem (β=0.485; p=0.000), and polymyxin B (β=0.401, p=0.005) was statistically significant. The prevalence of resistance to imipenem was 50% in January 2010 and 71.4% in December 2013; that of resistance to meropenem was 44.4% in January 2010 and 71.4% in December 2013, while the prevalence of resistance to polymyxin B was zero in January 2010 and 7.1% in December 2013. However, significantly decreasing resistance of *P. aeruginosa* to amikacin (β=-0.345; p=0.016) and piperacillin/tazobactam (β=-0.301, p=0.038) were observed (Table 2).

**TABLE 2:** Trends in the prevalence of resistance of *P. aeruginosa* to and consumption of antimicrobial agents of clinical interest in nine ICUs from January 2010 to December 2013.

Antimicrobial agent	**Trend in the prevalence of *P. aeruginosa* resistance**				Trend in antimicrobial agent consumption			
	β	p	Trend	KS significance	β	p	Trend	KS significance
Amikacin	-0.345	**0.016**	decreasing	0.974	0.793	0.000	increasing	**0.445**
Gentamicin	0.037	0.802	increasing	0.769	0.746	**0.000**	increasing	**0.845**
Ciprofloxacin	0.040	0.786	increasing	**0.966**	0.188	0.200	increasing	0.341
Imipenem	0.419	**0.003**	increasing	**0.927**	-0.875	**0.000**	decreasing	0.904
Meropenem	0.485	**0.000**	increasing	**0.754**	0.571	**0.000**	increasing	0.301
Cefepime	0.163	0.269	increasing	**0.470**	-0.825	**0.000**	decreasing	0.575
Ceftazidime	0.217	0.138	increasing	**0.967**	0,336	**0.020**	increasing	0.016
Piperacillin/ Tazobactam	-0.301	**0.038**	decreasing	0.673	0.245	0.094	increasing	**0.889**
Polymyxin B	0.401	**0.005**	increasing	0.010	0.625	**0.000**	increasing	0.210

**β coefficient:** indicates the association between a dependent variable and independent variables by a linear regression model. The significance level was set at 0.05. **KS significance**: indicates the significance of the Kolmogorov-Smirnov normality test. Variables were considered normal when KS>0.005.

It is noteworthy that 457 (48.7%) of the 939 isolates of *P. aeruginosa* were classified as MDR *P. aeruginosa* (PaMDR), with a higher prevalence in the years 2010 (51.2%) and 2013 (52.9%).

Consumption of amikacin (β=0.793; p=0.000), gentamicin (β=0.746; p=0.000), meropenem (β=0.571; p=0.000), ceftazidime (β=0.336; p=0.020), and polymyxin B (β=0.625; p=0.000) were significantly increasing. In contrast, consumption of imipenem (β=-0.875; p=0.000) and cefepime (β=-0.825; p=0.000) were significantly decreasing (Table 2).

In addition, significant associations were observed between the consumption of and resistance of *P. aeruginosa* to amikacin (β=-0.361; p=0.012), imipenem (β=-0.316; p=0.029), meropenem (β=0.327; p=0.023), and polymyxin B (β=0.351; p=0.014) (Table 3). 

**TABLE 3:** Association between the consumption of antimicrobial agents of clinical interest and the prevalence of *P. aeruginosa* resistance in nine ICUs from January 2010 to December 2013.

Antimicrobial agent	β	p	Impact	KS significance
Amikacin	-0.361	**0.012**	Negative	**0.774**
Gentamicin	0.082	0.579	Positive	**0.858**
Ciprofloxacin	-0.002	0.992	Negative	**0.881**
Imipenem	-0.316	**0.029**	Negative	**0.350**
Meropenem	0.327	**0.023**	Positive	**0.534**
Cefepime	-0.239	0.102	Negative	**0.399**
Ceftazidime	-0.199	0.176	Negative	**0.982**
Piperacillin/Tazobactam	-0.062	0.675	Negative	**0.953**
Polymyxin B	0.351	**0.014**	Positive	0.001

**β coefficient:** indicates the association between a dependent variable and independent variables by a linear regression model. The significance level was set at 0.05. **KS significance:** indicates the significance of the Kolmogorov-Smirnov normality test. Variables were considered normal when KS>0.005.

In this study, *P. aeruginosa* was the second most frequently isolated bacterium, representing 14.5% of the total isolates, which demonstrates the clinical importance of this microorganism. The results obtained are consistent with those of other studies, such as the one performed in ICUs of a public hospital in Ceará (Brazil), wherein *P. aeruginosa* represented 33.8% of the isolates^6^, and that performed in an ICU of Anesthesiology in Turkey, wherein the prevalence of *P. aeruginosa* was 14.29%^2^, demonstrating that the high prevalence of this microorganism is common in various countries.

The resistance profiles of *P. aeruginosa* against the tested antibiotics are presented in Table 1. It is noteworthy that Biswall et al. (2014), in India, reported similar resistance prevalence results for aztreonam (41.38%) and piperacillin/tazobactam (34.5%), as well as zero resistance to colistin (or polymyxin E). However, the results were different for amikacin (81.03%), gentamicin (81.03%), ceftazidime (70.68%), imipenem (18.9%), and meropenem (13.79%)^7^. Based on these data, there may be variations in microbial resistance profiles depending on the study site, which shows the importance of determining resistance profiles to be able to select the most appropriate antimicrobial therapy.

It is also worth noting that the prevalence of resistance of *P. aeruginosa* to imipenem and meropenem showed an increasing trend in the study period. These data concur with the results of a study carried out at a hospital in the state of Santa Catarina (Brazil), which indicated that resistance to carbapenems was increasing. Thus, in 2004, the resistance of *P. aeruginosa* to imipenem was 6.06%, increasing to 48.09% in 2008; for meropenem, the resistance increased from 6.89% in 2004 to approximately 20% in 2008^8^. Similarly, Xu et al. (2013) in a study performed at a hospital in China found increasing resistance to imipenem (β=4.620; p<0.001) and meropenem (β=4.624; p<0.001)^9^. Although carbapenems are one of the most potent classes of antimicrobials against *P. aeruginosa,* increasing resistance to these drugs is an endemic problem in several countries; therefore, the World Health Organization (WHO) considers the development of new drugs for this microorganism a priority^10^.

In addition, the present study showed a decreasing prevalence of resistance of *P. aeruginosa* to amikacin and piperacillin/tazobactam, which differs from the results of Xu et al. (2013), who showed that resistance to amikacin (β=0.488; p=0.332) and piperacillin/tazobactam (β=4.346; p<0.001) was increasing^9^.

Although the resistance of *P. aeruginosa* to polymyxin B was found to be low (0.9%) in the present study, resistance to this antibiotic was observed to increase (β=0.401, p=0.005). This result is worth mentioning as polymyxins are considered the last line of defense against gram-negative bacteria and have been classified by the WHO as critically important for human medicine^11^.

Among the isolates of *P. aeruginosa,* 48.7% were classified as MDR. This result is similar to the prevalence of 30.5% observed in a multicenter study of patients admitted to hospitals in Europe and the United States^4^. Similarly, a study carried out at a Spanish hospital indicated that 33.3% of *P. aeruginosa* isolates were classified as MDR^12^. It is also worth noting that in Brazil, Neves et al. (2010) reported a frequency of 23.1% of PaMDR, in a study carried out at the Clinics Hospital of Botucatu of the School of Medicine of the State of São Paulo^13^.

Therefore, it is important to note that the treatment options for PaMDR are limited. Most PaMDR are susceptible to polymyxins (polymyxin B and colistin). Aminoglycosides may be another alternative^3^.

Considering the consumption of antimicrobial agents, increasing tendency for the consumption of amikacin and gentamicin was observed in the present study. However, Vega et al. (2015) observed a decrease in the consumption (in DDD/100 patients-day) of amikacin (from 0.87 in 2008 to 0.31 in 2011) and an increase in the consumption of gentamicin (from 1.17 in 2008 to 10.67 in 2011) in a study carried out in an adult ICU in Argentina^14^. In another study, an increase in the consumption (in DDD/100 patients-days) of both amikacin (from 1.48 in 1999 to 1.91 in 2008) and gentamicin (from 5.02 in 1999 to 6.88 in 2008) was observed in ICUs in the Czech Republic^15^.

In the present study, a downward trend in imipenem consumption was identified, while meropenem consumption was increasing. In the study by Vojtová et al. (2011), increasing consumption of both imipenem (from 0.33 in 1999 to 1.60 in 2008) and meropenem (from 0.88 in 1999 to 3.56 in 2008) was observed^15^.

In addition, a decreasing trend in the consumption of cefepime was observed in the present study, whereas ceftazidime showed an increasing trend. In the study by Vega et al. (2015), the consumption (in DDD/100 beds-day) of cefepime and ceftazidime decreased between 2008 and 2011 from 10.19 to 1.48 and 2.2 to 0.25, respectively^14^.

Furthermore, a growing trend of consumption was found for polymyxin B, perhaps because of the increased prevalence of PaMDR in the study period and because polymyxins are currently the last option for monotherapy against resistant and PaMDR^3^, and are therefore extensively used for this purpose.

By analyzing the correlation between the prevalence of resistance to and the consumption of antimicrobial agents, we observed a negative association between the consumption of amikacin and imipenem and prevalence of *P. aeruginosa* resistance to these antibiotics. However, even though the consumption of amikacin increased during the study period, we cannot conclude that the decrease in the prevalence of resistance was only due to the possible effectiveness of the antimicrobial since it is known that the decrease in prevalence of bacterial resistance also depends on other factors^1^ such as increased surveillance, campaigns to combat the spread of resistance, and changes in microbial resistance profiles.

It is still noteworthy that a positive impact and association between the consumption of meropenem and polymyxin B and the prevalence of resistance of *P. aeruginosa* to these antibiotics were detected. Xu et al. (2013) obtained similar results for meropenem (β=1.241; p<0.001) but different results for imipenem (β=1.238; p<0.001)^9^. As for polymyxin B, the result obtained is worrisome as polymyxins are the last option for monotherapy against resistant and PaMDR^3^. However, it is important to note that in the present study, *P. aeruginosa* showed a low resistance profile to polymyxin B.

The present study has the following limitations: (i) the presented consumption data were general data for the nine selected ICUs, not specific data for patients with *P. aeruginosa* infection; and (ii) other factors that influence resistance were not considered, although antimicrobial consumption is one of the most important factors^1^.

Thus, a prospective study using specific antimicrobial consumption data and data from each patient with *P. aeruginosa* infection, as well as the analysis of all associated factors and/or resistance influencers, may help better establish the correlations between bacterial resistance and antimicrobial consumption, as well as between bacterial resistance and other associated factors and/or resistance influencers.

In the present study, we determined the prevalence of resistant and MDR isolates of *P. aeruginosa*, as well as the levels of antibiotic consumption in ICUs of a university hospital in São Paulo (Brazil). The results of this work emphasize the importance of monitoring the consumption of antimicrobial agents and combating the increase of microbial resistance, including multidrug resistance.
